# Bacterial Isolates and Their Antimicrobial Susceptibility Profile in Osteomyelitis Patients: An Experience From a Tertiary Care Center in a Hilly Area of Uttarakhand

**DOI:** 10.7759/cureus.44263

**Published:** 2023-08-28

**Authors:** Dimple Raina, Uneza Husain, Pavneesh Kumar, Ajay K Pandita, Nidhi Negi

**Affiliations:** 1 Microbiology, Shri Guru Ram Rai Institute of Medical and Health Sciences, Dehradun, IND; 2 Microbiology, Government Doon Medical College, Dehradun, IND; 3 Orthopaedics, Government Doon Medical College, Dehradun, IND; 4 Community Medicine, Shri Guru Ram Rai Institute of Medical and Health Sciences, Dehradun, IND

**Keywords:** surgery, infection, marrow, bone, osteomyelitis

## Abstract

Osteomyelitis, an infection related to bone and bone marrow, is very diverse in its pathophysiology and clinical presentation; hence, it is considered one of the most difficult-to-treat infections. The present study is aimed at assessing the microbiological profile of osteomyelitis and related antimicrobial susceptibility patterns in patients attending a tertiary care center in Uttarakhand, India, over a period of one year (January to December 2019). In aerobic culture, 58/105 (55.2%) bacterial isolates were detected. In addition, *Staphylococcus aureus* was the most common isolate, and amongst Gram-negative bacilli, most isolates that grew on culture were *Escherichia coli *(22.4%). Out of 21 *S. aureus *isolates, methicillin resistance was detected in nine [9/21 (42%)] cases, which is a matter of concern. Hence, proper training and application of antimicrobial stewardship are the need of the hour so that clinicians can initiate targeted therapy as early as possible.

## Introduction

The term osteomyelitis is derived from the Greek words “osteon” (meaning bone) and “muelinos” (meaning marrow); hence, it refers to the serious infection related to bone and bone marrow [1]. The disease can occur due to local spread from a contaminated nidus following trauma or surgery, secondary to vascular insufficiency, especially in diabetic patients, or via a hematogenous route [2]. The patients may present with signs and symptoms, such as pain, fever, chills, swelling, redness around the infected site, purulent discharge, fistula, and sinus [3,4]. Acute osteomyelitis usually presents two weeks after bone infection and is characterized by inflammatory changes in the bone. Chronic osteomyelitis, on the other hand, presents six or more weeks after bone infection and is characterized by bone necrosis and/or formation of a large area of de-vascularized dead bone called “sequestrum” [5]. Osteomyelitis is very diverse in its pathophysiology and clinical presentation; hence, it is considered one of the most difficult-to-treat infections. The most common cause of hematogenous osteomyelitis in children and adults is *Staphylococcus aureus* [6,7]. Other causes include coagulase-negative *Staphylococcus*; *Streptococcus* (beta-hemolytic); Enterococci; aerobic Gram-negative bacilli (GNB) such as *Escherichia coli*, *Pseudomonas* species, and *Enterobacter* species; and anaerobic GNB such as *Peptostreptococcus*, *Bacteroides*, and *Clostridium* species. In immunocompromised patients, *Mycobacterium tuberculosis*, nontuberculous mycobacteria, *Candida* species, and fungi, such as *Blastomyces*, *Coccidioides*, *Cryptococcus*, and *Aspergillus*, are implicated as pathogens, though they are less commonly encountered [7,8]. Regarding the diagnosis of osteomyelitis, bone biopsy with histopathologic examination and tissue culture is considered the gold standard [3]. Radiographic imaging such as plain radiographs, magnetic resonance imaging (MRI), and technetium-99 bone scintigraphy are essential components of the evaluation of such patients [7]. With the emergence of multidrug-resistant microorganisms such as methicillin-resistant *S. aureus*, the treatment of the infection has become challenging in the present time. The present study aimed mainly at assessing the microbiological profile of osteomyelitis and related antimicrobial susceptibility patterns in patients attending a tertiary care center in a hilly area of Uttarakhand, India.

## Materials and methods

The study was conducted at the Shri Guru Ram Rai Institute of Medical and Health Sciences, a tertiary care hospital in Dehradun, India. A total of 105 patients (indoor/outdoor), presented to the orthopedics department with complaints of pain, fever, swelling, and redness around the infected site, purulent discharge, and fistula or sinus and were provisionally diagnosed with osteomyelitis from January 1 to December 31, 2019, were included in the study. Bone biopsied material, tissue, pus, or discharge from the infected site were collected and sent to the department of microbiology for processing (microscopic examination, culture, and antimicrobial susceptibility testing). All samples were inoculated onto MacConkey agar and blood agar plates. The plates were incubated aerobically for 24-48 hours. Isolates were identified, and antimicrobial susceptibility testing was done by an automated Vitek 2 Compact system (bioMerieux, Inc., Durham, NC, USA). Minimum inhibitory concentrations (MICs) were interpreted as per the latest Clinical and Laboratory Standards Institute (CLSI) guidelines. The information collected from patient records included gender, age, infecting organisms, and susceptibility pattern. The present study is retrospective in nature, so written consent from the patients was not taken, and before data analysis, their identifying particulars were removed to maintain anonymity.

## Results

**Figure 1:**
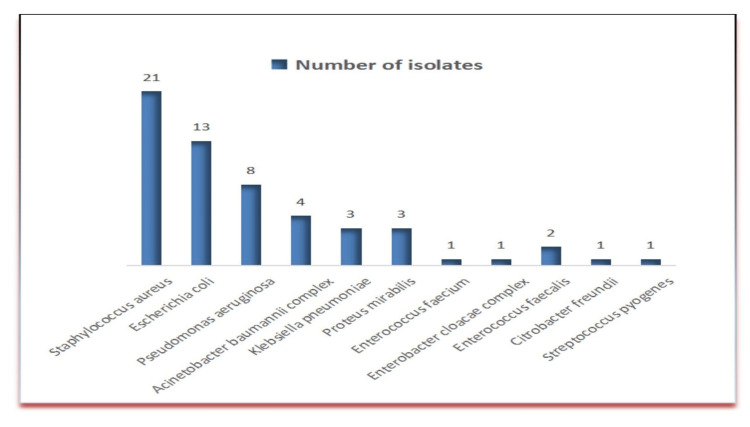
Number of bacterial isolates in culture from cases of osteomyelitis (organism-wise distribution)

From a total of 105 specimens collected from osteomyelitis cases at the orthopedics department, 58 (55.2%) came out to be positive by culture (and/or microscopy). The number of culture-negative specimens was found to be 47 (44.8%) [including four specimens in which contamination with skin flora was suspected and hence was not considered as significant, and repeat testing with proper specimen collection was done that later turned out to be negative]. Males (86/105, 81.9%) outnumbered females with a majority of cases belonging to the 40-60 years of age group. *S. aureus* was the predominant organism isolated in the majority of cases [21/58 (36.2%)]. Out of 21 *S. aureus* isolates, methicillin resistance was detected in nine [9/21 (42%)] cases. Amongst GNB, the most common organism isolated was *Escherichia coli*, which was detected in 13/58 (22.4%) cases. Others were *Pseudomonas aeruginosa*, *Acinetobacter baumannii*, *Klebsiella pneumoniae*, *Proteus mirabilis*, *Enterococcus* spp., *Streptococcus pyogenes*, *Enterobacter cloacae*, and *Citrobacter freundii*. The number of bacterial isolates in osteomyelitis cases is summarized in Figure 1. The antimicrobial susceptibility profile of the most commonly isolated organism in our study is summarized in Table 1.

**Table 1:** Antimicrobial susceptibility pattern in frequently isolated pathogens in cases of osteomyelitis *In case of colistin, I% is considered as S% because there is no S breakpoint available S, susceptible; I, intermediate; R, resistant

Antimicrobial agent	Staphylococcus aureus		Escherichia coli		Pseudomonas aeruginosa		Acinetobacter baumannii	
	Susceptible (S%)	Not susceptible (I + R%)	Susceptible (S%)	Not susceptible (I + R%)	Susceptible (S%)	Not susceptible (I + R%)	Susceptible (S%)	Not susceptible (I + R%)
Oxacillin	58	42	-	-	-	-	-	-
Erythromycin	52	48	-	-	-	-	-	-
Clindamycin	95	5	-	-	-	-	-	-
Vancomycin	100	0	-	-	-	-	-	-
Linezolid	100	0	-	-	-	-	-	-
Gentamicin	84	16	80	20	-	-	28	72
Ciprofloxacin	10	90	50	50	63	37	-	-
Levofloxacin	57	43	-	-	54	46	71	29
Ampicillin	-	-	20	80	-	-	-	-
Ceftriaxone	-	-	34	66	-	-	-	-
Ceftazidime	-	-	-	-	55	45	57	43
Piperacillin-Tazobactam	-	-	70	30	63	37	42	58
Imipenem	-	-	90	10	72	28	42	58
Meropenem	-	-	90	10	72	28	42	58
Amikacin	-	-	100	0	63	37	28	72
Tigecycline	-	-	100	0	-	-	71	29
Minocycline	-	-	-	-	-	-	71	29
Colistin*	-	-	100	0	63	37	71	29

## Discussion

The incidence of the devastating disease osteomyelitis is estimated to be 21.8 cases per 100,000 person-years, though the actual picture is difficult to predict [9]. As per published reports, nearly one in 675 hospital admissions/year or approximately 50,000 osteomyelitis cases occur annually in the United States [7]. The disease is said to be more common in young children and older adults, although no age group is spared. The most common sites of hematogenous osteomyelitis in adults are vertebral bodies, followed by long bones, pelvis, and clavicle [3]. In the case of children, mainly long bones are affected. Males are more commonly affected than females, as evident from our study and other published literature on osteomyelitis [7].

The current literature suggests a high percentage of chronic osteomyelitis cases being culture-negative ranging between 28% and 50% [10-12]. Our study being retrospective, we could not differentiate between acute and chronic osteomyelitis cases, but culture positivity (55.2%) was similar to other studies published previously [11,12]. In general, Gram-negative organisms as a group were isolated predominantly compared to the Gram-positive organism group in our study, similar to the study done by Vijayakumar et al. [13]. Discussing the specific organism, *S. aureus* was the most common isolated organism in our study. This finding was supported in a review done by Urish et al., in which the authors mentioned that across all mechanisms leading to osteomyelitis, *S. aureus* is the most common organism [9,14-16]. *S. aureus* is known to express receptors, called adhesins, which can help in the adhesion with the bone matrix, including laminin, fibronectin, bone sialoglycoprotein, and collagen [7]. Collagen-binding adhesin permits its attachment to bone cartilage, while fibronectin-binding adhesin plays an important role in the attachment of bacteria to surgically implanted devices in bone [7]. A noteworthy point in the context of *S. aureus* is that it can survive intracellularly after being internalized by cultured osteoblasts [2,7]. Out of 21 *S. aureus* isolates, methicillin resistance was detected in nine [9/21 (42%)] cases, which is a matter of concern. Hence, proper training and application of antimicrobial stewardship are the need of the hour so that clinicians can initiate targeted therapy as early as possible.

Although challenging, the disease is usually managed by a multidisciplinary approach, including antibiotics, infected tissue debridement, reaming, bone troughing, bone fenestration, Masquelet technique, segmental resection with callus distraction, bone grafting, and even amputation depending on the stage of the disease [17]. In a recent study by Fang et al., vancomycin along with superparamagnetic iron oxide nanoparticles was used in a rat disease model for osteomyelitis treatment. They inferred from the study that magnetic nanoparticles increased the efficiency of the treatment due to the generation of hyperthermia by the particles, leading to the eradication of the biofilm [18]. The treatment should be aimed at preventing the progression of this catastrophic disease and its sequelae.

Limitations of the present study are as follows:

- Our study being retrospective, we could not differentiate between acute and chronic osteomyelitis.

- Correlation of clinical findings with the corresponding etiology could not be ascertained.

- Due to the shorter duration of the study (one year), we would like to suggest that future researchers explore this topic further before reaching any concrete conclusion in the context of the etiology of the disease or its susceptibility pattern.

## Conclusions

Osteomyelitis is a heterogeneous disease with a vivid presentation, which can lead to devastating complications if left untreated. Methicillin resistance is increasing, which is a matter of concern. Even if the clinical diagnosis of osteomyelitis is obvious, the microbiological workup for etiological diagnosis of cases of osteomyelitis is still not a routine practice in many hospitals, which needs to be improved.
